# Evaluation of Pre-operative Biopsy, Surgical Procedures and Oncologic Outcomes in Upper Tract Urothelial Carcinoma (UTUC)

**DOI:** 10.3389/fsurg.2021.790738

**Published:** 2021-11-25

**Authors:** Florestan J. Koll, Eva Meisenzahl, Bernhard Haller, Philipp Maisch, Florian Kirchhoff, Thomas Horn, Jürgen E. Gschwend, Sebastian C. Schmid

**Affiliations:** ^1^Department of Urology, Rechts der Isar Medical Center, Technical University of Munich, School of Medicine, Munich, Germany; ^2^Department of Urology, University Hospital Frankfurt, Frankfurt, Germany; ^3^Institute of Medical Informatics, Statistics and Epidemiology, Technical University of Munich, Munich, Germany; ^4^Department of Urology, University of Ulm, Ulm, Germany

**Keywords:** adjuvant chemotherapy, nephroureterectomy, segmental ureteral resection, ureteroscopy, upper tract urothelial carcinoma, UTUC

## Abstract

**Purpose:** Discordance between pre-operative biopsy and final pathology for Upper Tract Urothelial Carcinoma (UTUC) is high and optimal management remains controversial. The aim of this study is to evaluate the accuracy of pre-operative biopsy, to identify prognostic factors and to evaluate the effect of adjuvant chemotherapy on survival and oncologic outcome in UTUC.

**Methods:** We analyzed records of patients receiving surgical treatment for UTUC. Pathology of pre-operative biopsy was compared to surgical specimen. We used Kaplan-Meier method to estimate survival probabilities and Cox's proportional hazards models to estimate the association between covariates and event times. Primary endpoint was overall survival (OS). A matched-pair analysis was performed to evaluate the effect of adjuvant chemotherapy.

**Results:** 151 patients underwent surgical treatment (28% open, 36% laparoscopic, 17% robotic radical nephroureterectomy; 14% segmental ureteral resections and 5% palliative nephrectomy) for UTUC and were included in the analysis. Upstaging from <pT1 in endoscopic biopsy to ≥pT1 in final pathology occurred in 61% of patients and upgrading from low-grade to high-grade occurred in 30% of patients. Five-year OS was 59.5%. In the univariate Cox-regression model pathological stage, grade, lymphovascular invasion and positive surgical margins were associated with OS. Matched pair analysis for stage (<pT3; ≥pT3; pN+) and age revealed a significant survival benefit for adjuvant chemotherapy (HR 0.40, 0.14–0.77, *p* < 0.018) in this cohort.

**Conclusion:** UTUC is often underestimated in pre-operative biopsy, and it is associated with significant mortality. Pathological stage and grade, lymphovascular invasion and lymph node metastases are predictors of oncologic outcome and survival.

## Introduction

Upper urinary tract urothelial carcinoma (UTUC) is a rare disease that accounts for around 5% of all urothelial cancers (1). At the time of diagnosis around 60% of UTUC are invasive tumors (2).

For the diagnostic evaluation imaging, mainly computed tomography urography, urinary cytology and diagnostic ureteroscopy (URS) are used (3). The pre-operative grading is unreliable and associated with high rates of upgrading at final pathology (4, 5). Direct staging using URS is not possible since biopsies are only taken from the superficial ureteral layer.

The standard of care for invasive UTUC is radical nephroureterectomy (RNU) with excision of an ipsilateral bladder-cuff, and lymph node dissection (LND) (3), which can be performed as open, laparoscopic or robotic surgery. Segmental ureteral resection can be an alternative for low-risk tumors, that cannot be removed completely endoscopically and for high-risk tumors when renal function preservation is necessary (6, 7). However, data about outcome for different surgical approaches is sparse. Because of high recurrence rates, mostly retrospective studies have investigated perioperative chemotherapy. While studies on neoadjuvant chemotherapy (NAC) only consist of small cohorts leading to insufficient survival data, adjuvant chemotherapy has stronger evidence to improve survival in locally advanced or lymph node (LN) positive disease and is recommended by the EAU-Guidelines (3, 8–10).

To address these diagnostic and therapeutic challenges in management of UTUC we carried out a retrospective analysis to evaluate the accuracy of pre-operative biopsy, the outcome of surgical approaches and the survival-rates.

## Patients and Methods

### Study Population

We analyzed patients with UTUC receiving surgical treatment from 2008 to 2019 at Medical Center Rechts der Isar, Technical University of Munich, Germany. The local ethics committee approved the present study, which was conducted according to local and national regulations. We included patients receiving surgical procedures like RNU or segmental ureteral resections. Patients were excluded if the main tumor was in the bladder and RNU was part of the cystectomy.

Patient data, medical history, radiologic, pathologic and operative findings were gathered from medical records and independently reviewed by two authors. Follow-up and survival status were collected from medical charts, tumor registry Munich and from patient information.

We defined the overall survival (OS) as main endpoint of interest, which was defined as time interval between surgery and death. Secondary endpoints were disease-free survival (DFS), defined as time interval between surgery and death due to UTUC or recurrence and cancer-specific survival (CSS) defined as time interval between surgery and tumor-related death.

Further endpoints were the rate of upgrading and upstaging from pre-operative biopsy to final pathology and to define differences between the surgical procedures in regard to oncologic outcome and complications. We aimed to evaluate the influence of perioperative chemotherapy on OS and DFS. Patients receiving adjuvant chemotherapy were compared to surgery alone using a matched-pair analysis.

### Statistical Analysis

We performed descriptive statistics on all data. For the correlation between pre-operative biopsy or cytology and final pathology we calculated sensitivity and specificity.

Kaplan-Meier method was used to estimate and illustrate survival probabilities. We used univariable Cox's proportional hazards models to estimate the hazard ratio (HR) and corresponding 95% confidence interval for covariates for OS and DFS. All tests were two-tailed, and a significance level of α = 5% was used. Statistical analyses were performed using R Statistical Software (Version 4.0) and R Studio (Version 1.3.959). For patients receiving adjuvant chemotherapy we performed a matched-pair analysis using propensity-score matching with the MatchIt package (version 3.0.2). Patients were matched in ratio 1 treated to 2 controls using the setting “optimal” considering post-operative tumor stage (<pT3; ≥ pT3; pN+) and age as matching characteristics. A jitter-plot for distribution of propensity scores is shown in Supplementary Figure 1.

## Results

### Patient Characteristics

Overall, we included 151 patients with UTUC. Surgical procedures included 42 (28%) open, 55 (36%) laparoscopic and 26 (17%) robotic RNU as well as 21 (14%) segmental ureteral resections and seven (5%) palliative nephrectomies. The median age of the study population was 72 years (IQR 67–78) and 97 patients (64%) were male. In final pathology 74 patients (49%) had pathological stage pT2 or higher and 108 patients (72%) had high grade urothelial carcinoma, including G2-tumors (Supplementary Table 1).

### Correlation of Biopsy and Cytology With Final Pathology

In total, 110 patients (73%) had biopsy prior to surgery. Ninety-eight patients (65%) had ureterorenoscopic biopsy, 11 patients (7%) had CT-controlled biopsy and one patient had sonography-controlled biopsy.

Patients with URS biopsy were analyzed for the concordance of pre-operative and final pathology. Patients with neoadjuvant chemotherapy were excluded. Biopsy showed non-invasive UTUC (pT0/pTa/pTis) in 77 patients (82%) and invasive UTUC (pT1) in 17 patients (18%). In final pathology 62 patients (66%) had invasive cancer (≥pT1). The sensitivity for detection of invasive UTUC was 24% and the specificity was 94%. Fifty-seven (61%) patients were upstaged and 35 patients (37%) had the same stage comparing biopsy and final pathology.

**Figure 1:**
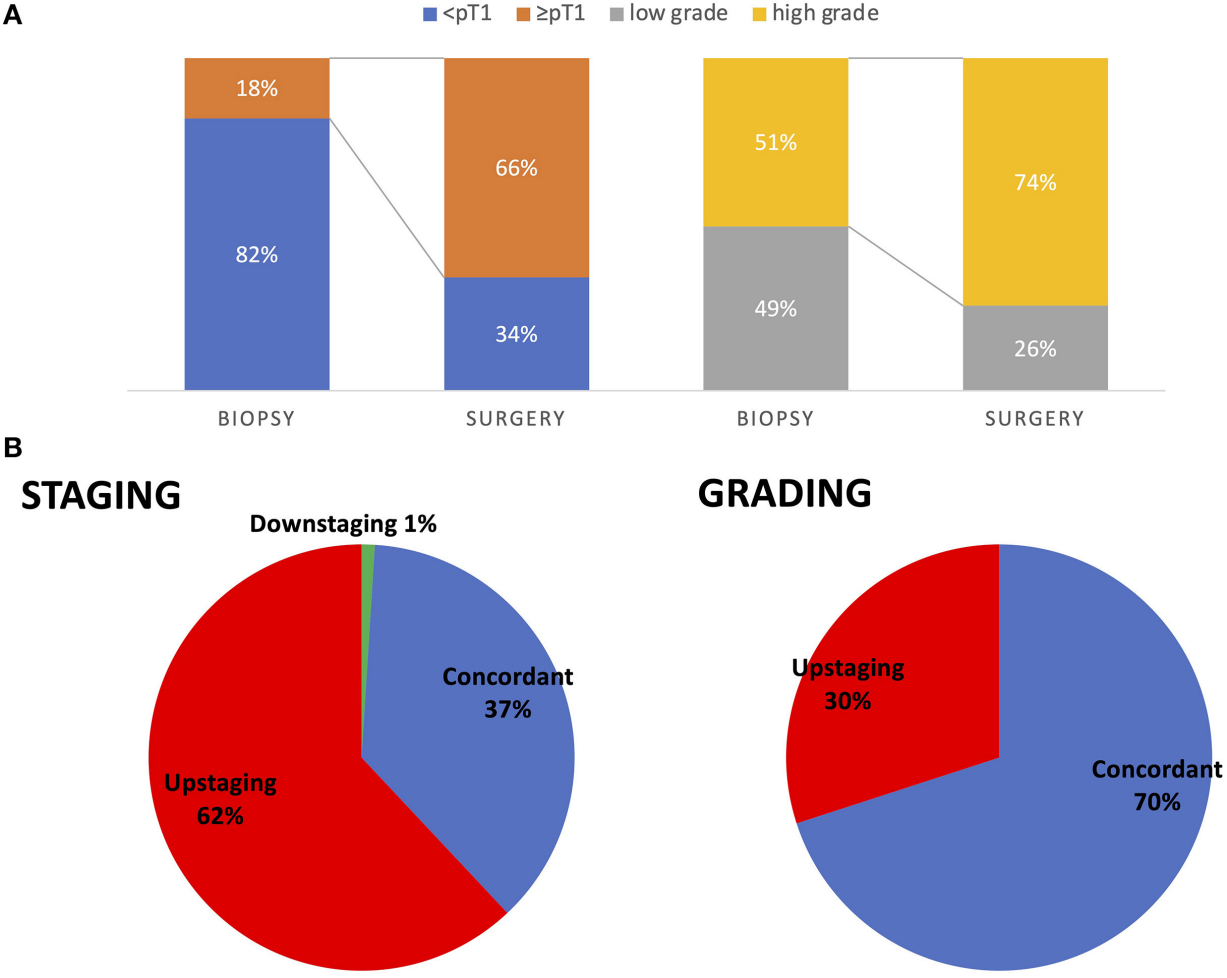
Comparison of pre-operative biopsy to final pathology for pathologic staging and grading in all patients **(A)**. Rate of patients being upstaged from pre-operative biopsy to final pathology. Seventy percent (64 patients) had concordant pathologic grading in URS biopsy and final pathology and 30% (28 patients) were upstaged from low grade to high grade. Sixty-two percent (58 patients) had pathologic upstaging from URS to final pathology, 37% (35 patients) had concordant pathologic stage. One patient had downstaging of pTa tumor in URS and no malignant residual tumor in RNU **(B)**.

Detecting high-grade carcinoma with URS had a sensitivity of 69%. None of the high-grade carcinomas detected in biopsy were downgraded in the final pathology, but 28 (30%) of low-grade carcinomas were upgraded (Figure 1).

Selective urine cytology during URS was obtained in 50 patients. Selective cytology was not usable in 4 patients (8%), negative in 10 patients (20%) with 2 patients having low-grade tumor and 8 having high-grade tumor in final pathology. Of 36 patients (72%) with positive cytology (including atypical cells) 9 patients (18%) had low-grade and 24 (48%) had high-grade (including G2) tumor in final pathology. Sensitivity for detection of high-grade carcinoma was 75% and for detection of invasive tumor (≥pT1) 76%, respectively.

### Surgical Procedures

Forty-two patients (28%) received open, 55 patients (36%) laparoscopic and 26 patients (17%) robotic RNU; 21 patients (14%) had segmental ureteral resections and seven patients (5%) palliative nephrectomy (Table 1).

**Table 1:** Patient and surgical characteristics with 30-Day post-operative complications specified for all complications and Clavien–Dindo grade ≥3 stratified by surgical technique.

		**Open RNU**	**Robotic RNU**	**Laparoscopic RNU**	**Segmental** **ureteral resections**
**Number of patients**		**42 (29%)**	**26 (18%)**	**55 (38%)**	**21 (15%)**
Median age (IQR)		72 (67–78)	74 (69–78)	72 (67–79)	71 (65–76)
Final pathology	≤pT1	19 (45%)	13 (50%)	30 (55%)	15 (67%)
	pT2	5 (12%)	2 (7%)	7 (13%)	2 (10%)
	pT3/pT4	18 (43%)	11 (42%)	18 (33%)	4 (19%)
Grading post-operative	Low grade	7 (17%)	4 (15%)	17 (31%)	9 (43%)
	High grade	31 (74%)	21 (81%)	37 (67%)	12 (57%)
	NA; ypT0	4 (9%)	1 (4%)	1 (2%)	0
Lymph node status	pN0	17 (40%)	16 (62%)	18 (33%)	11 (52%)
	pN+	11 (26%)	3 (12%)	4 (7%)	4 (19%)
	NA; pNx	14 (33%)	7 (27%)	33 (60%)	6 (29%)
Resection margins	R0	33 (79%)	24 (92%)	50 (91%)	19 (90%)
	R1/R2/Rx	9 (21%)	2 (8%)	5 (9%)	2 (10%)
Lymphadenectomy	No	15 (36%)	7 (27%)	34 (62%)	5 (24%)
	Yes	27 (64%)	19 (73%)	21 (38%)	16 (76%)
	Median No of LN removed (IQR)	11.5 (6–17)	7 (5–13)	5 (1.3–9)	8 (5–13)
Localization	Kidney pelvis	32 (76%)	13 (50%)	36 (65%)	0
	Ureter	4 (10%)	7 (27%)	9 (16%)	21 (100%)
	Multiple	6 (14%)	6 (23%)	10 (18%)	0
Bladder cuff excision		29 (69%)	24 (92%)	51 (92%)	17 (81%)
**Complications**
**Any**		**13 (31%)**	**2 (8%)**	**18 (33%)**	**8 (38%)**
	Bleeding	4 (9%)	0	7 (13%)	2 (10%)
	Infection	2 (5%)	0	3 (5%)	2 (10%)
	Bowel-perforation or ileus	3 (7%)	0	3 (5%)	0
	Cardiovascular	2 (5%)	2 (8%)	0	1 (5%)
	Other (lymphocele, chylascitis)	1 (2%)	0	5 (9%)	3 (14%)
**Clavian-Dindo ≥3**		**6 (14%)**	**2 (8%)**	**8 (15%)**	**6 (29%)**
	Bleeding	1 (2%)	0	4 (7%)	2 (10%)
	Infection	0	0	1 (2%)	2 (10%)
	Bowel-perforation or ileus	3 (7%)	0	3 (5%)	0
	Cardiovascular	1 (2%)	2 (8%)	0	0
	Other (lymphocele, chylascitis)	0	0	0	2 (10%)

*RNU, radical nephroureterectomy*.

*Total number of patients per column are in bold*.

More patients with advanced cancer received open RNU (43% pT3/pT4-tumors, 21% positive resection margins and 26% positive LN status) compared to laparoscopic RNU (33% pT3/pT4-tumors, 9% positive resection margins and 7% positive LN status). In 26 patients that received robotic RNU, 43% had pT3/pT4-tumor, but with lower risk of positive resection margins (7%) and lower rate of positive LN status (12%) compared to open surgery. More patients with robotic RNU than with open RNU received LND (73 vs. 63%), but the median number of LN removed was lower in robotic RNU (7 vs. 12).

We observed positive or unknown resection margins in 18 (13%) patients, which was associated with open RNU in 9 cases, advanced tumors (13 patients with pT3/4) and lymph node metastases (7 patients).

Ten Patients have had cystectomy before RNU of which eight had open RNU, one had laparoscopic RNU and one had segmental ureteral resection. This also contributes to the low rate of 69% of patients getting bladder cuff excision with open RNU.

### Complications

Nine (16%) of the 55 laparoscopic RNU and one of the 26 robotic RNU were converted to open surgery.

We detected 13 (31%) and 18 (33%) complications up to 30 days post-operatively and six (14%) and eight (15%) severe complications in open and laparoscopic RNU, respectively, but only two (8%) complications in robotic RNU. In open RNU one patient (2%) had a severe bleeding, three patients (7%) had ileus or bowel-perforation and one patient (2%) had severe cardiovascular complication. In laparoscopic RNU four patients (7%) had severe bleeding, one patient (2%) had an infectious complication and three patients (5%) had ileus or bowel-perforation, whereas in robotic RNU two patients (8%) had cardiovascular complications (Table 1). We noticed a higher number of complications in segmental ureteral resections, specifically two (10%) with bleeding, two (10%) with infections and three (14%) with lymphocele. Of seven patients with ileal interposition, one had severe bleeding and one had severe infection leading to revision surgery. Of two patients with end-to-end anastomosis, one developed lymphocele that needed drainage. And of 12 patients that had ureteroneocystostomy, three patients had revision surgery due to bleeding, wound dehiscence and lymphocele, respectively.

### Adjuvant Chemotherapy

**Figure 2:**
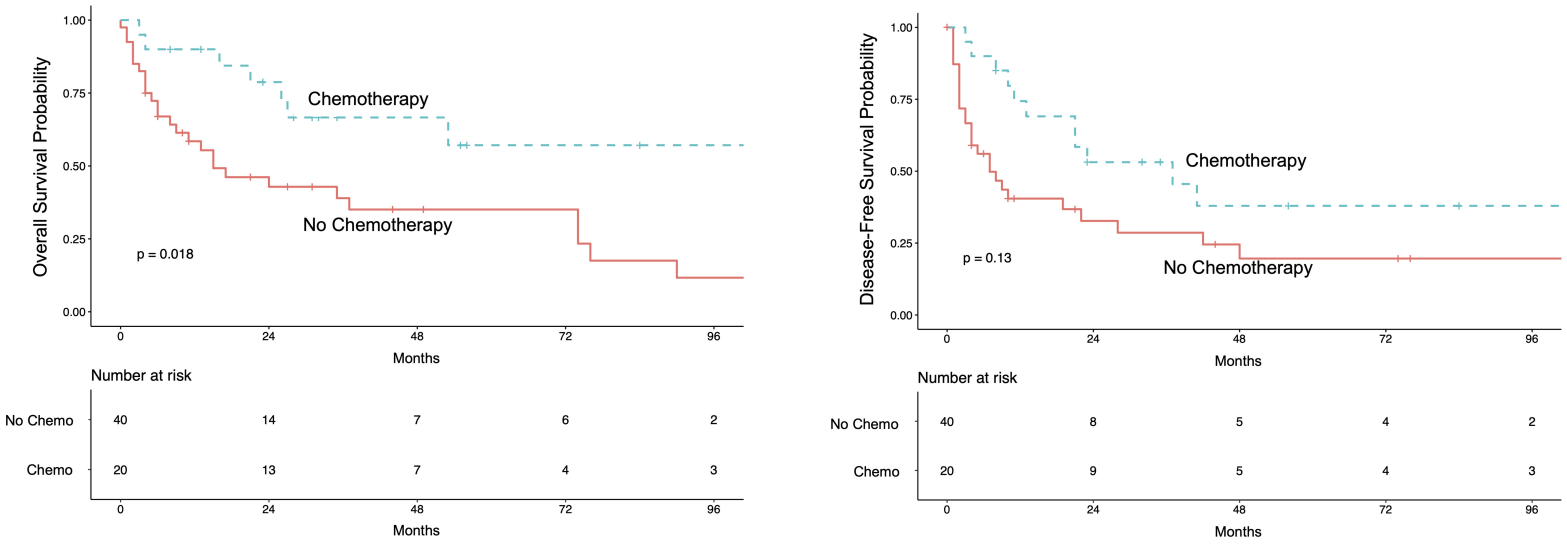
Kaplan-Meier curve for overall survival (left) and disease-free survival (right) for the matched pair analysis for patients receiving adjuvant chemotherapy. Patients were matched in ratio 1 treated to 2 controls for post-operative tumor stage (<pT3; ≥pT3; pN+) and age. In the adjuvant chemotherapy-group 11 patients received Gemcitabine/Cisplatin; 4 patients received Gemcitabine/Carboplatin; 5 patients received other therapies.

We analyzed 20 patients receiving adjuvant treatment after RNU. Patients were matched in a ratio 1 treated to 2 controls considering post-operative tumor-stage (<pT3; ≥pT3; pN+) and age. Five-year OS for patients receiving adjuvant treatment was 57% (95%CI 37–89%) vs. 35% (95%CI 22–56%) for patients without adjuvant treatment (Figure 2). A survival-analysis stratified for tumor- and lymph node status showed a survival benefit for adjuvant chemotherapy with 1 year OS of 90% for pT3/4-tumors (95%CI 73–100%) and 87% for pN+ (95%CI 67–100%) compared to patients without chemotherapy of 72% for pT3/4-tumors (95%CI 73–100%) and 37% for pN+ (95%CI 20–66%) (log-rank for chemo vs. no chemo *p* = 0.01). This points out that especially those patients with lymph node metastases benefit most from adjuvant chemotherapy (Supplementary Figure 2). A multivariate Cox-regression model adjusting for post-operative tumor-stage and age revealed a benefit for adjuvant chemotherapy with a HR of 0.40 (95%CI 0.14–0.77; *p* < 0.018).

### Survival

**Figure 3:**
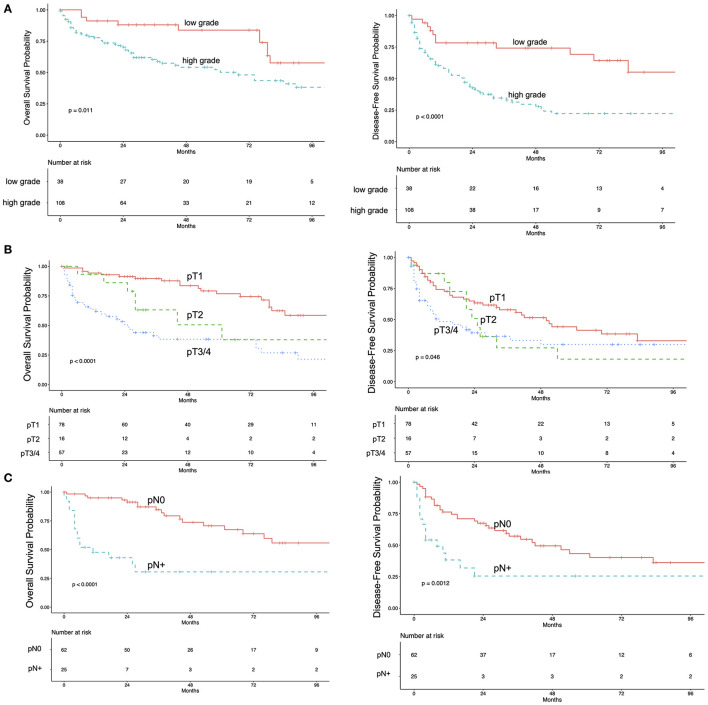
Kaplan-Meier estimates for overall survival (left) and disease-free survival (right) in 151 patients with upper tract urothelial carcinoma stratified by pathologic grade **(A)** pathologic T-Stage **(B)** and lymph node status **(C)**.

The median follow-up was 68 months (IQR 29–105). During follow-up 66 events were observed, 39 patients had tumor-related death. The 5-year OS rate was 59.5% (95%CI 51–69%). The 5-year OS-rate for pTa/pTis/pT1 was 77% (95%CI 66–89%), 51% (95%CI 28–92%) for pT2, 38% (95%CI 26–56%) for pT3/pT4 and 30% (95%CI 16–59%) for pN+, regardless of the pT-status. Five-year OS for patients with high-grade and low-grade UTUC was 52% (95%CI 42–64%) and 83% (95%CI 72–98%), respectively (Figure 3). Overall DFS-rates for 1 year and for 5 years were 66% (95%CI 58–74%) and 36% (95%CI 28–46%), respectively. The 5-year CSS was 69% (95%CI 61–78%).

In the univariate Cox-regression model higher pathological stage, high-grade disease, positive LN status, lymphovascular invasion and positive surgical margins were associated with decreased OS (Table 2). Bladder-cuff resection and post-operative instillation of Mitomycin C were significantly associated with increased OS.

**Table 2:** Univariate Cox-regression model for overall survival.

**Variable**		***n***	**Hazard ratio (95%CI)**	***p***
Age		151	1.07 (1.04–1.11)	***<0.0001***
Sex	Female vs. male	54	1.10 (0.66–1.83)	0.712
ECOG	≥2 vs. 0, 1	6	3.14 (1.23–8.03)	***0.0167***
Grading	High-grade vs. low-grade	94	2.27 (1.18–4.35)	***0.011***
Tumor stage	≥pT2 vs. < pT2	74	3.45 (2.05–5.79)	***<0.0001***
Lymph node metastases	pN+ vs. pN0	25	5.13 (2.57–10.24)	***<0.0001***
Resection margins	R1/R2/Rx vs. R0	22	6.37 (3.58–11.36)	***<0.0001***
Cis	Cis vs. No Cis	25	1.77 (0.98–3.21)	0.06
Lymphovascular invasion	Yes vs. No	29	2.96 (1.35–6.48)	***0.0067***
Localization	Ureter vs. kidney pelvis vs. multiple	39	0.62 (0.32–1.22)	0.116
		24	0.84 (0.42–1.66)	0.819
Lymphadenectomy	Yes vs. No	86	0.97 (0.51–1.82)	0.751
Hydronephrosis	Yes vs. No	75	1.19 (0.73–1.94)	0.487
Mitomycin C instillation	Yes vs. No	26	0.30 (0.09–0.96)	***0.0428***
Bladder cuff excision	Yes vs. No	122	0.37 (0.22–0.63)	***0.00022***

*Bold and italics values indicate significant differences with p-values <0.05*.

We observed cancer recurrence in 79 patients. Median time from surgery to recurrence or tumor related death were 28 months (IQR 21–50). Seventeen patients (11%) had local recurrence, ten patients (7%) had recurrence on the other side, 34 patients (23%) had recurrence in the bladder and 33 patients (22%) developed distant metastases. Bladder cancer was diagnosed in 73 patients (48%) and occurred in 31 patients before surgery of UTUC, in 12 patients concurrently and in 30 patients after surgery of UTUC.

## Discussion

This study investigates treatment of UTUC in a representative cohort of 151 patients. Limitations of our study are the single institution data collection, the retrospective design and the lack of randomization.

Inadequacy of biopsy for diagnosis of UTUC is a known issue (5, 11, 12). Dev et al. described upgrading in 41% and upstaging in 63% from URS biopsy to RNU specimen (12). Smith et al. found reclassification in 43% from low-grade, non-invasive disease to high-grade and/or invasive disease in repeated URS biopsies (11). Our results with an upstaging rate of 61% and an upgrading rate of 30% point out the difficulty of a correct pre-operative diagnosis. Lower tract cytology has a poor sensitivity for detecting UTUC, but selective upper tract cytology reaches sensitivity between 71 and 81% and specificity of over 90% for high-grade disease (13–15). We noticed sensitivity of 75% for detecting high-grade UTUC with selective cytology, but also 25% of high-grade tumors had negative cytology. Imaging using CT-urography has a high sensitivity of about 97% and specificity of 93% and the secondary sign of hydronephrosis is associated with advanced disease and poor oncological outcome (3, 16). Thus, a combination of diagnostic modalities can improve the pre-operative prediction of grade and stage (13, 15, 17).

Open RNU with bladder-cuff excision is the standard of care for high-risk UTUC (3). In a systematic review evaluating laparoscopic RNU the authors conclude that oncologic outcome might be reduced in patients with locally advanced tumors (18) and thus should not be performed in invasive tumors or when LN metastases are suspected (3). When comparing the different surgical procedures, we noticed a higher number of advanced cancers receiving open surgery compared to a minimal invasive approach. Especially the high rates of positive resection margins in 24% and LN metastases in 26% of patients with open RNU indicate advanced tumors. Bladder-cuff excision was less frequently performed in the open RNU due to previous cystectomy, non-organ confined or palliative tumors. Selection criteria for surgical procedures were not standardized and patients with suspected larger tumors and/or LN metastases received preferably open surgery in most of the cases. Furthermore, laparoscopic RNU was performed until 2015 while robotic RNU was performed from 2015 onwards. Interestingly, in 26 patients that received robotic RNU we observed a similar rate of high-grade UTUC (81%) and pT3/pT4 tumors (43%) but with lower risk of positive resection margins (7%) and lower rate of positive LN status (12%), compared to open surgery. The rate of LND and the median yield of LNs removed was significantly higher in robotic vs. laparoscopic LND (73 vs. 38% and 7 LNs vs. 5 LNs). A retrospective study on 2,631 patients by Kenigsberg showed that patients with laparoscopic RNU less likely underwent LND (19 vs. 35%) and had a lower median lymph node yield (3 vs. 4) compared to robotic RNU (19). These results are in line with ours, although the rate and yield of LND was much higher in our study. According to our data, robotic RNU had a high rate of R0-resections, bladder-cuff excisions and LND with acceptable complication rates (19, 20) and superior outcome. However, this observation may be also related to a selection bias but is at least hypothesis generating.

In general, 57% of patients had LND with a median of seven LNs removed, leading to 17% of patients with positive LN status, which was significantly associated with decreased survival. The rate of patients having LND and the median number of LNs removed was higher than in previous, even larger trials reporting LND (2, 21, 22). Neither LND itself, nor the number of LNs removed were associated with survival rates. As lymph node status is a main trigger for adjuvant chemotherapy, the regional LN dissection is of importance, while the optimal extent and field is still undefined.

In our small cohort with patients receiving segmental ureteral resection, a complete tumor and proper LN resection was possible, but with a comparatively high number of complications (6, 7).

We observed positive or unknown resection margins in 18 patients (13%) receiving RNU. The rate of positive R1-findings for minimal invasive approaches were comparable to previous series reporting laparoscopic and robotic RNU (19, 23). But the rate of 21% patients with R1/Rx-findings in patients receiving open RNU seems to be higher and was mostly associated with advanced tumor stage and/or positive LN status. Since positive resection margins are strongly associated with poor outcome, we suggest that in patients with advanced tumors which might not be completely respectable, a neoadjuvant chemotherapy could be discussed to achieve downstaging and better conditions for surgery.

For many years the recommendations for adjuvant chemotherapy in advanced UTUC were based on retrospective and non-randomized studies and meta-analyses showing a benefit for adjuvant chemotherapy (3, 9, 24). In 2020 Birtle et al. published results of the POUT trial, a phase 3, randomized controlled trial analyzing adjuvant chemotherapy with gemcitabine and carboplatin/cisplatin in UTUC including pT2-T4 pN0-3 cM0 or any pT pN1-3 cM0. In this trial, the estimated 3-year DFS and metastasis-free survival was significantly increased in comparison to standard surveillance (8).

Our findings regarding adjuvant chemotherapy are in line with published data and show a significant survival benefit for OS and a small but not significant benefit for DFS and CSS. Despite the propensity-score matching for age and tumor stage, our analysis holds a risk of selection bias, since kidney function and comorbidities were not included.

In regard to survival, the 5-year OS of 59.5% confirms the high mortality risk of this disease. The oncologic outcome with 5-year CSS of 69% is within the range reported in previous studies reporting 5-year CSS of 61%-76% (2, 21, 25, 26). Despite radical surgery with RNU, 22% of patients developed distant metastases and 26% of patients had tumor-related death.

Our findings suggest that pathological parameters from the RNU-specimen are important for patient risk stratification. These include grading, tumor stage, LN metastases, resection margins and lymphovascular invasion. These factors have been described before to be associated with outcome after RNU. The influence of tumor grade in the two-tiered grading system with low- and high-grade tumors has been described as controversial. Corresponding to the results of the largest cohort of RNU by Margulis et al. we found grading in the RNU specimen to be predictive for OS, CSS and DFS (2). Older series did not show tumor grade to be an independent predictor of oncologic outcome (26, 27). We and others have identified lymphovascular invasion as a predictor for decreased survival (2, 26), but so far its impact on further therapy decisions like adjuvant chemotherapy is not defined (3).

Concluding our experience and the results of this study, we suggest that RNU with bladder cuff excision can be performed as open or robotic surgery depending on the experience of the surgent. But a lymphadenectomy should be component of the surgery in high-risk disease and include LND on the affected side of the ureter and retroperitoneal LND for higher ureteral and renal pelvis tumors. Further randomized prospective trials are needed to compare open vs. robotic regarding oncologic outcome. The application of adjuvant chemotherapy has been proven to prolong DFS for patients with muscle invasive UTUC. Thus, platin-based combination chemotherapy is the standard of care for patients with pT2–pT4 or LN-positive tumors treated with RNU with curative intent.

## Conclusion

We conducted a study to evaluate the pre-operative pathological diagnostic, surgery techniques, predictors of survival and perioperative chemotherapy. UTUC is often underestimated in pre-operative biopsy and it is associated with significant mortality. Predictors of survival like lymph node and tumor status should be taken into account for further therapy decision and to guide adjuvant chemotherapy.

## Data Availability Statement

The raw data can be provided upon request to the corresponding author/s.

## Ethics Statement

The study was conducted according to the guidelines of the Declaration of Helsinki, and approved by the Ethics Committee of the Technical University Munich (291/21 S-SR).

## Author Contributions

FK: conceptualization, methodology, formal analysis, investigation, writing—original draft, data curation, formal analysis, and visualization. EM: investigation, data curation, formal analysis, and visualization. BH: writing—review and editing and formal analysis. PM and TH: writing—review and editing. FK: writing—review and editing and data curation. JG: writing—review and editing and supervision. SS: project administration, conceptualization, methodology, writing—review and editing, and supervision. All authors contributed to the article and approved the submitted version.

## Conflict of Interest

The authors declare that the research was conducted in the absence of any commercial or financial relationships that could be construed as a potential conflict of interest.

## Publisher's Note

All claims expressed in this article are solely those of the authors and do not necessarily represent those of their affiliated organizations, or those of the publisher, the editors and the reviewers. Any product that may be evaluated in this article, or claim that may be made by its manufacturer, is not guaranteed or endorsed by the publisher.
