# Supplementary material for: Evaluation of Pre-operative Biopsy, Surgical Procedures and Oncologic Outcomes in Upper Tract Urothelial Carcinoma (UTUC)
Source: Front Surg. 2021 Nov 25;8:790738. doi: 10.3389/fsurg.2021.790738 (PMC8655158; doi:10.3389/fsurg.2021.790738)
Supplement: Supplementary file 1 [file Data_Sheet_1.PDF]

Supplementary Material

Evaluation of Preoperative Biopsy, Surgical Procedures and Oncologic Outcomes in Upper Tract Urothelial Carcinoma (UTUC)

Supplementary Table 1: Clinicopathological characteristics of the 151 patients with UTUC analyzed in this study. IQR = interquartile range; ECOG = Eastern Cooperative Oncology Group; ASA = American Society of Anaesthesiologists

**Variable**		**n (151)**
**Median Age (IQR)**		72 (67 – 78)
**Gender**	Male	97 (64%)
	Female	54 (36%)
**ECOG**	<2	109 (72%)
	≥2	6 (4%)
	NA	36 (24%)
**ASA**	1	7 (5%)
	2	91 (60%)
	3	39 (26%)
	NA	14 (9%)
**Localization**	Kidney pelvis	88 (58%)
	Ureter	39 (26%)
	Multiple	24 (16%)
**Final tumor stage**	≤pT1	78 (52%)
	pT2	16 (11%)
	pT3	46 (30%)
	pT4	11 (7%)
**Lymph node metastases**	pN0	62 (41%)
	pN+	25 (17%)
	pNx	64 (42%)
**Final grading**	Low grade	38 (26%)
	High grade	108 (72%)
**Smoker**	No	71 (47%)
	Yes (incl. Ex-smoker)	62 (41%)
	NA	18 (12%)

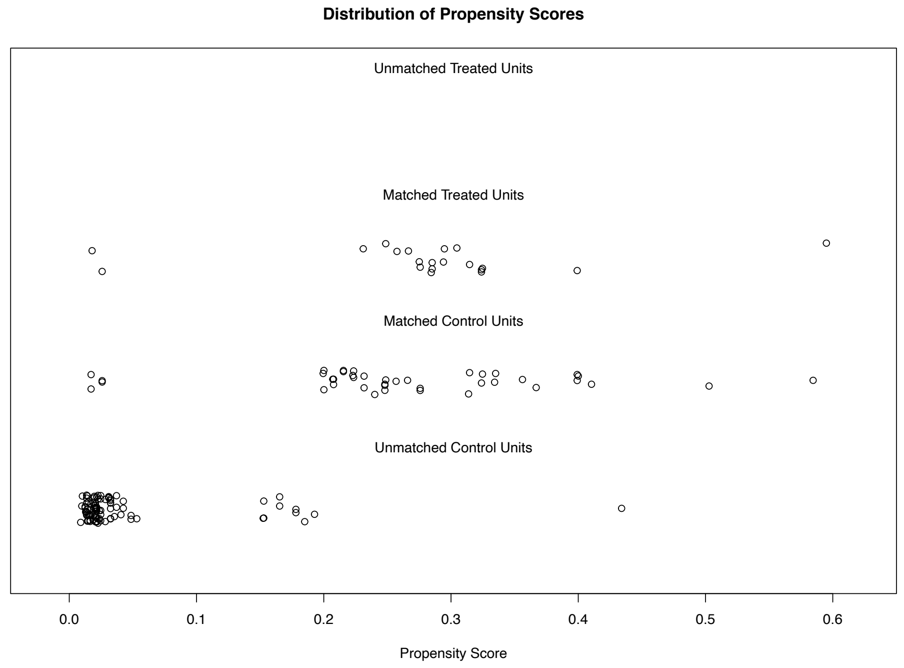

Supplementary Figure 1: Jitter plot for propensity score matching. Patients were matched in ratio 1 treated to 2 controls using the setting "optimal" considering postoperative tumor stage (<pT3; ≥ pT3; pN+) and age as matching characteristics.

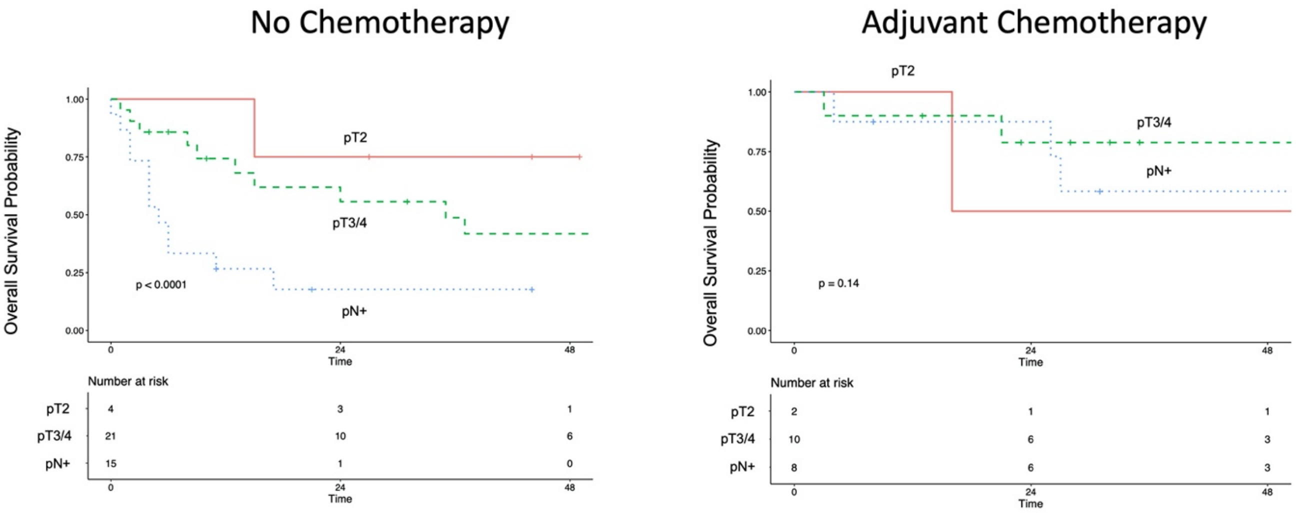

Supplementary Figure 2: Stratified survival analysis of the matched groups according to tumor and lymph node stage for overall survival. Log rank between the adjuvant chemotherapy and surveillance group p=0.01.
